# Renewable Fabric Surface-Initiated ATRP Polymerizations: Towards Mixed Polymer Brushes

**DOI:** 10.3390/nano10030536

**Published:** 2020-03-17

**Authors:** Wojciech Raj, Alessandro Russo, Yaoming Zhang, Julien Chapelat, Joanna Pietrasik

**Affiliations:** 1 Lodz University of Technology, Institute of Polymer and Dye Technology, Stefanowskiego 12/16, 90-924 Lodz, Poland; wojciech.raj@dokt.p.lodz.pl (W.R.); yaomingzhang@licp.cas.cn (Y.Z.); 2 Cemex Research Group AG, Römerstrasse 13, 2555 Brüggbei Biel, Switzerland; alessandro.russo@cromogenia.com (A.R.); julien.chapelat@cemex.com (J.C.); 3 Key Laboratory of Solid Lubrication, Lanzhou Institute of Chemical Physics, Chinese Academy of Sciences, Lanzhou 730000, China

**Keywords:** mixed polymer brushes, surface modification, surface thermal rearrangement

## Abstract

A totally new approach in the synthesis of mixed polymer brushes tethered on polyamide (PA) surfaces is presented herein. As a proof of concept, two types of homopolymers were synthesized in sequential surface-initiated atom transfer radical polymerization (SI-ATRP) reactions: poly(methyl methacrylate)/poly((2-dimethylamino)ethyl methacrylate) and polystyrene /poly((2-dimethylamino)ethyl methacrylate). The ATRP initiator was immobilized on the surface through PA chain-end groups in two subsequent steps, separated by homo-polymerizations. The amount of the PA chains’ end groups available on the modified surface was tuned by the thermal rearrangement of the surface.

## 1. Introduction

Tethering polymer chains to a surface is a very useful synthetic strategy, as it allows extensive tuning of the surface properties simply by adjusting the composition, functionality or architecture of the tethered polymer chains, called polymer brushes. In general, the parameters, such as molecular weight (MW), molecular weight distribution (MWD) as well as the number of chains per surface area, which influences the polymer chains conformation and thickness of the polymer layer, are considered crucial aspects for determining the properties of the final materials [1,2,3]. The ability to design and tune the surface properties allow the polymer brushes to be widely used in a broad range of applications, not only as stabilizing colloidal systems [4,5] but also in antibiofouling [6], organic electronics [7], biosensing [8], and as stimuli-responsive materials [9,10]. Mixed polymer brushes that contain two different types of polymers are very special types of polymer brushes, as they allow a combination of various selected properties of homopolymers into one system [11].

There are a few synthetic strategies for the synthesis of polymer brushes [12]. In the “grafting to” approach, the pre-synthesized polymer chains which contain an appropriate functional group located either at the chain end or along the polymer backbone are immobilized on the surface through the interactions between the available functional groups on the polymer and the surface [13]. Through this approach, well-defined polymer brushes can be obtained; however, a high grafting density of high molecular weight polymer brushes is not achievable [14]. On the other hand, the “grafting from” approach allows reaching a high grafting density of tethered polymer brushes, because the polymerization starts from the immobilized initiators [15] thereby allowing concurrent chain growth. Running the polymerization with good control makes it possible to tune the number of chains growing from the surface.

The characterization of MW and MWD is a challenge for cleaved polymer brushes from low surface area materials. To overcome this problem, free/sacrificial initiators are usually introduced to the system and then the polymerization from the surface and in the solution proceed in parallel. In such a case, the free polymer is an analogue of the polymer grafted from the surface [16,17,18], although some studies have postulated the kinetic of polymer growth on the surface differs [19]. Several polymerization mechanisms, including ionic, ring opening, and radical polymerization, have been applied to the synthesis of polymer brushes but reversible deactivation radical polymerization (RDRP) [20,21,22,23,24] is the most common one, as it allows for the growth of the polymer brushes in a controlled way while the broad applicability of a radical polymerization is maintained. Surface-initiated atom transfer radical polymerization (SI-ATRP) has been extensively used, mostly due to the straightforwardness of the initiator attachment to various types of the substrates [13]. According to our best knowledge, the majority of current synthetic routes are based on formation of a single-(co)polymer functionalized substrate. Only a few reports demonstrate the possibility of double or multi-use functional substrates. A “print, erase, and reprint” approach was reported for the rewritable polymer brushes on glass and silicon substrates based on click chemistry [25,26]. Zhou et al. demonstrated the repeatable surface modification based on an initiator-embedded polystyrene sheet that did not require specific surface chemistry for the initiator immobilization [27].

Herein, we demonstrate a facile method of sequential immobilization of ATRP-initiating groups on the surface of polyamide (PA) fibers by the thermal rearrangement of the polymer support and tethered chains. First, certain amounts of ATRP initiators were immobilized on the PA fibers followed by the first SI-ATRP step. Next, through temperature treatment of the composite fiber, the polymer chains within the fiber gained some mobility, leading to the creation of new functional groups on the surface of the fibers. Therefore, a second series of initiator immobilization and polymerization was able to be conducted. The PA was chosen because the amine chain end groups are easily modified directly by the ATRP initiator. Furthermore, the broad scope of PA’s potential applications (e.g., textile, packaging, electronics) requires very specific PA surface properties. As a proof of concept, two types of homopolymers were synthesized in sequential SI-ATRP reactions: poly(methyl methacrylate) (PMMA)/poly((2-dimethylamino)ethyl methacrylate) (PDMAEMA) and polystyrene (PS)/poly-((2-dimethylamino)ethyl methacrylate) (PDMAEMA). Also, the capability to tune the antibacterial properties of the formed hybrid fibers by incorporation of well-defined PDMAEMA chains was demonstrated. This work demonstrates a new strategy of surface modification for polymer fibers with wide application potential. Moreover, a new concept of mixed brush synthesis is introduced, as this kind of dual surface modified polymer brushes are usually obtained through tethering dual functionalized Y initiators on the substrate, followed by using two different polymerization mechanisms or through a grafting approach by using the mixture of two homopolymers [11,18,28,29,30,31,32,33,34]. Herein, the described novel procedure is very simple, does not require complicated synthetic procedures, neither for initiator synthesis nor the surface treatment, and can be accommodated to any polymeric supports containing chain-end functional groups. 

## 2. Materials and Methods 

### 2.1. Materials

2-Bromo-2-methylpropionyl bromide (BIBB, 98%, Sigma–Aldrich, Poznan, Poland), ethyl 2-bromo-2-methylpropionate (EBIB, 98%, Sigma–Aldrich, Poznan, Poland), 2,2,6,6-tetramethyl-1-piperidinyloxy (TEMPO, 98%, Sigma–Aldrich, Poznan, Poland), 4,4’-dinonyl-2,2’-bipyridine (dNbpy, 97%, Sigma–Aldrich), copper (I) bromide (CuBr, 99.999%, Sigma–Aldrich, Poznan, Poland), copper (II) bromide (CuBr_2_, 99.999%, Sigma–Aldrich, Poznan, Poland), anisole (reagentplus^®^, 99%, Sigma–Aldrich, Poznan, Poland), acetone (99.5% pure p.a. basic, POCH) were used as received. 2-(Dimethylamino)ethyl methacrylate (DMAEMA, 98%, Sigma–Aldrich, Poznan, Poland) and methyl methacrylate (MMA, 99 %, Alfa Aesar, Kandel, Germany) were purified by passing through a column filled with basic alumina. Knitted fabric (100% polyamide PA; grammage 60g/m^2^) was purchased from Zaklad Produkcji Dziewiarskiej Waldemar Rosinski in Lodz (Lodz, Poland). Enriched nutrient broth was purchased from Zaklad Enzymow I Peptydow BTL sp. z o.o.

### 2.2. Modification of PA Knitted Fabric

#### 2.2.1. PA Modification with ATRP Initiator: PA-Br

Polyamide (PA) knitted fabric (5.0 g), N-methyl-2-pyrrolidone (50.0 mL), and triethylamine (4.3 mL, 30.99 mmol) were added to a round-bottom flask and then immersed in an ice bath. After the flask cooled, BIBB (4.6 mL, 37.2 mmol) was added dropwise over ~30 min. The reaction mixture was stirred for 24 h at room temperature. The modified fibers were removed from the reaction mixture and washed several times with acetone to obtain the PA fabric with tethered ATRP initiators, PA-Br. 

#### 2.2.2. Grafting of Poly(Methyl Methacrylate) from PA Surface via SI-ATRP: PA-*g*-PMMA_164_-Br

A Schlenk flask was charged with the initiator-modified knitted fabric (PA-Br) (376.2 mg), copper(I) bromide (16.1 mg, 0.113 mmol), copper(II) bromide (16.8 mg, 0.075 mmol), and 4,4’-dinonyl-2,2’-dipyridyl (152.9 mg, 0.375 mmol). Next, the flask was degassed by purging with argon for 45 min at room temperature. The MMA (10.0 mL, 93.9 mmol), anisole (5.0 mL), and ethyl 2-bromo-2-methylpropionate EBIB (28 µL, 0.188 mmol) were degassed separately and added to the reaction flask via a syringe. The flask was placed in an oil bath preheated to 80 °C for 5 h. The polymerization was stopped by opening the flask and exposing the reaction mixture to air. The obtained hybrid knitted fabric, PA-*g*-PMMA_164_-Br, was separated from the reaction mixture, washed several times by extraction with acetone, dried in a vacuum oven, and used for further analysis. The free PMMA prepared in the solution was diluted with THF, purified by passage through a column filled with neutral alumina, precipitated, and dried in a vacuum oven.

#### 2.2.3. Deactivation of PA-*g*-PMMA_164_-Br Chains Ends with Tributyltin Hydride (Bu_3_SnH): PA-*g*-PMMA_164_-H

The PMMA-modified knitted fabric PA-*g*-PMMA_164_-Br (193.8 mg), CuBr (50 mg, 0.7 mmol), Bu_3_SnH (0.55 mL, 4.2 mmol), and 4,4’-dinonyl-2,2’-dipyridyl (165 mg, 2.1 mmol) were put into a Schlenk flask. Next, the flask was degassed by purging argon for 45 min at room temperature, and then degassed anisole (10.0 mL) was added via a syringe. The reaction to replace the bromide –Br end group by Bu_3_SnH was started after putting the flask into an oil bath heated to 80 °C and was complete after 3 h. The resulting knitted fabric PA-*g*-PMMA_164_-H was washed a few times with acetone.

#### 2.2.4. Rearrangement of PA-*g*-PMMA_164_-H

The PA-*g*-PMMA_164_-H (149.1 mg) and deionised water (100 mL) were put into a beaker and heated under water boiling conditions. The knitted fabric was removed from the water after 1 h and dried in a vacuum oven for 24 h.

#### 2.2.5. Modification PA-*g*-PMMA_164_-H with an ATRP Initiator: PA-*g*-[(PMMA_164_-H), Br]

The PA-*g*-PMMA_164_-H knitted fabric (149,1 mg), N-methyl-2-pyrrolidone (25.0 mL), and triethylamine (0.34 mL, 2.45 mmol) were added to a flask immersed in an ice bath. Once the contents of the flask were cooled down, 2-bromo-2-methylpropionyl bromide (0.36 mL, 2.95 mmol) was added dropwise over ~30 min. The reaction mixture was kept at room temperature overnight. The modified fibers were removed from the reaction mixture and washed several times with acetone and dried in vacuum oven overnight.

#### 2.2.6. Synthesis of Mixed-Polymer Brushes: PA-*g*-[(PMMA_164_-H)-*mixed brushes*-PDMAEMA_63_-Br]

The modified PA-*g*-[(PMMA_164_-H), Br] knitted fabric (95.6 mg), copper(I) bromide (14.2 mg, 0.099 mmol), copper(II) bromide (22.1 mg, 0.099 mmol), and 4,4’-dinonyl-2,2’-dipyridyl (161.7 mg, 0.396 mmol) were added to a Schlenk flask. The flask was then degassed by purging the argon for 45 min at room temperature. The DMAEMA (10.0 mL, 59.35 mmol), acetone (5.0 mL), and ethyl 2-bromo-2-methylpropionate EBIB (29.0 µL, 0.198 mmol) were degassed separately and added via a syringe to the reaction flask. The flask was placed in an oil bath heated to 60 °C for 5 h. The resulting dual grafted knitted fabric was separated from the reaction mixture, washed by extraction with acetone, and used for further analysis. Free PDMAEMA dissolved in THF was purified by passage through a chromatography column filled with neutral alumina, precipitated, and dried in the vacuum oven.

#### 2.2.7. Loading Gold Precursor HAuCl_4_ onto PA-*g*-[(PMMA_164_-H)-*mixed brushes*-PDMAEMA_63_-Br]: AuNPs@ PA-*g*-[(PMMA_164_-H)-*mixed brushes*-PDMAEMA_63_-Br]

A round-bottom flask was charged with the dual functional knitted fabric (150 mg) and gold chloride solution in deionized water (200 mg/dL, 5.0 mL). The mixture was kept at room temperature for 2 h; next, the fabric was removed, washed by extraction with water, and dried under vacuum.

### 2.3. Characterization of Surface Modified PA Knitted Fabric

#### 2.3.1. Nuclear Magnetic Resonance Characterization

Nuclear magnetic resonance (^1^H NMR) was used to determine the monomer conversion during the polymerization. The ^1^H NMR spectra were recorded on a Bruker ADVANCE DPX 250 MHz instrument (Bruker, Karlsruhe, Germany), using CDCl_3_ or DMSO-d_6_ as a solvent. 

#### 2.3.2. Gel Permeation Chromatography Characterization

Gel permeation chromatography (GPC) was used to determine the molecular weights and molecular weight distributions of the free polymer synthesized in solution. The GPC measurements were carried out using a Wyatt instrument (Wyatt, Dernbach, Germany) equipped with two Perfect Separation Solutions (PSS) columns and one guard column (GRAM Linear (10 mμ, *M_n_* between 800 Da–1,000,000), light scattering (LS), and differential refractometer (RI) detectors (Wyatt, Dernbach, Germany). The measurements were conducted in DMF with 50 mmol LiBr as eluent at a flow rate of 1 mL/min. Either polystyrene (PS) or poly(methyl methacrylate) (PMMA) standards were used for calibration, depending on the polymer composition (PS from *M_p_* = 1306 Da to *M_p_* = 1,210,000 Da; PMMA from *M_p_* = 960 Da to *M_p_* = 1,568,000 Da). 

#### 2.3.3. Fourier Transform Infrared Spectroscopy Characterization

Fourier transform infrared spectroscopy (FTIR) was used to characterize the presence of specific chemical groups in the materials. The FTIR spectra were obtained in the range of wavenumber from 4000 to 650 cm^−1^, doing 32 scans using a FTIR Nicolet 6700 spectrophotometer and OMNIC 3.2 software (Thermo Scientific Products: Riviera Beach, FL, USA). The ATR accessory equipped with a single reflection diamond ATR crystal on ZnSe plate was used for all the analysis.

#### 2.3.4. Elemental Analysis 

The Schöniger method was used for elementary analysis to quantitatively determine the amount of bromine atoms. The samples were burned and then titrated with the standard solution of Hg(ClO_4_)_2_).

#### 2.3.5. Scanning Electron Microscopy–Energy-Dispersive Spectroscopy Characterization, SEM-EDS

The morphology and composition of fabric was investigated using a S-4700 scanning electron microscope HITACHI (Tokyo, Japan), equipped with an energy-dispersive spectrometer (Thermo-Noran, Madison, WI, USA). The sample was sputter coated by gold prior to analysis. 

#### 2.3.6. Contact Angle Characterization

The contact angle (CA) was measured to determine the character of the surface before and after modification. A 50 μL drop of distilled water was placed by a micro-syringe on top of the knitted PA. The images were registered immediately using a camera coupled with the computer.

#### 2.3.7. Measurement of Bacteriostatic Properties

The potential anti-bacterial activity of the textile with the as-prepared gold nanoparticles was estimated by analysis of the resistance for *Escherichia coli*. The medium was prepared as follows: Nutrient broth (15 g/L) and agar (15 g/L) were mixed in distilled water and the pH was adjusted to 6.8 by the addition of a mixture of 0.1 M NaOH and 0.1 M CH_3_COOH, and then the medium was sterilized at 121 °C for 15 min. Afterwards, the medium was used to inoculate 1% *v*/*v* of *Escherichia coli* (Gram-negative) which was activated for one day in the nutrient broth medium (15 g/L) within 0.01% *v*/*v* of microbial. Finally, inoculated sterile medium was poured into Petri dishes in which round textile samples with a diameter of 1 cm were placed. After the solidification of the medium, two discs were incubated at 37 °C for 24 h. All the experiments were performed under sterile conditions.

## 3. Results and Discussion

A new procedure for the modification of a PA fabric by growing a mixture of brushes of PMMA/PDMAEMA and PS/PDMAEMA from its surface in the presence of a sacrificial initiator is presented herein. Detailed studies of two homopolymers’ synthesis were carried out to establish the most efficient route on how to tether two different types of polymers to the same surface using the same polymerization mechanisms. Atom transfer radical polymerization (ATRP) was used as the polymerization method. The fibers, with diameters in the micrometers range, were treated as flat surfaces, because the fibers’ dimensions were much larger in comparison to the dimensions of a single polymer chain. Moreover, the very low surface area of such materials makes the characterization process of polymer brushes grown from the fibers’ surfaces quite challenging. This is because, in many cases, it is impossible to grow a sufficient concentration of the polymer chains that could be detached quantitatively from the bare surface for MW and MWD analysis. Therefore, polymerizations were carried out in the presence of a sacrificial initiator in solution, assuming the polymer that grows from the surface and in the solution have the same MW and MWD [35].

The ATRP-initiating groups were introduced onto the surface of the fibers through the PA amine groups, assuming they were the chain end groups of PA, by using 2-bromo-2-methylpropionyl bromide. According to a previous report, an acid bromide could also interact with amide bonds of PA [36]. The bromine content on the modified surface was estimated through elemental analysis (EA), and was measured to be 0.19% wt in this case. This value was considered the lower range of the detection limitation of EA. Therefore, the amount of the initiator attached to the surface was negligible, and the molar ratios among all reagents were calculated based only on the concentration of the added free initiator. Methyl methacrylate and styrene were polymerized using the following molar ratios of the reagents: MMA(St)/EBIB/CuBr/CuBr_2_/dNbpy = 500(300)/1/0.6(0.7)/0.4(0.3)/2. Both polymerizations displayed good control forming polymers with narrow molecular weight distribution. The procedures for St polymerization are given in the Supplementary Materials. The theoretical DP of the grafted and free PMMA and PS was determined by ^1^H NMR based on monomer conversion calculated for the reaction mixture (Figures S1–S4). The GPC results of the PMMA and PS synthesized in solutions are shown in Table 1 and Figure S5. The modified textiles were weighted before and after polymerization and careful cleaning with acetone; the mass increased by 4 ± 0.2 mg and 7.3 ± 0.2 mg (corresponding to 1.05% wt; 1.69% wt) for PA-*g*-PMMA_164_-Br and PA-*g*-PS_66_-Br, respectively. On the contrary, polymerization from untreated textile did not cause any changes in its weight as well as in FTIR spectrum. 

**Figure 1:**
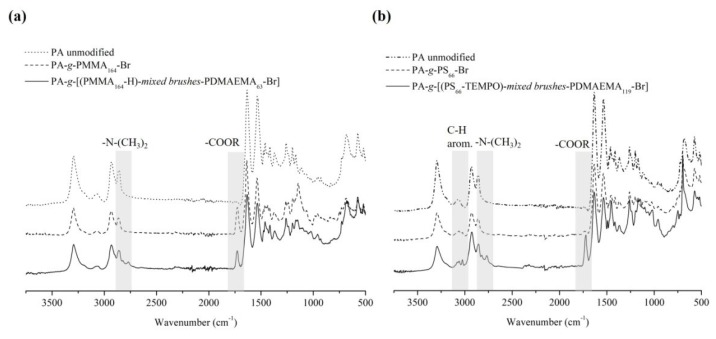
FTIR spectra obtained for PA fibers as well as for fibers modified with tethered (**a**) PMMA and PDMAEMA; PA-*g*-[(PMMA_164_-H)-*mixed brushes*-PDMAEMA_63_-Br] or (**b**) PS and PDMAEMA; PA-*g*-[(PS_66_-TEMPO)-*mixed brushes*-PDMAEMA_119_-Br] chains.

The successful grafting of PMMA and PS from PA fibers was verified by FTIR spectroscopy. Figure 1 presents the spectra of unmodified PA fibers and PMMA or PS modified fibers. The presence of the strong peak at 1735 cm^−1^ is assigned to the occurrence of an ester carbonyl group from poly(methyl methacrylate) [37] (Figure 1a). In the case of PS, numerous peaks characteristic for PS overlap with the PA support (i.e., the peak at the range of 1600 cm^−1^) associated with the presence of aromatic protons of styrene units, except the weak peak at 3100–3000 cm^−1^ which is also associated with aromatic protons of styrene [38] (Figure 1b).

**Figure 2:**
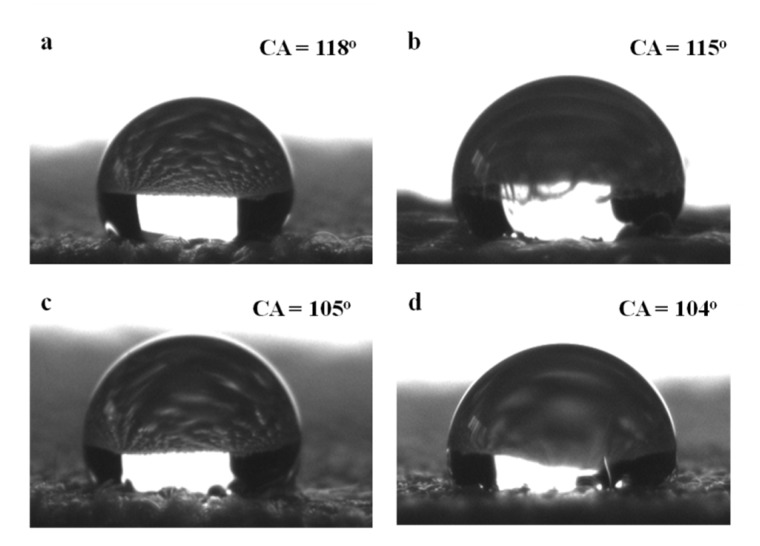
Images of water drops deposited on the PA surface modified with tethered polymers chains (**a**) PA-*g*-PS_66_-Br, (**b**) PA-*g*-PMMA_164_-Br, (**c**) PA-*g*-[(PS_66_-TEMPO)-*mixed brushes*-PDMAEMA_119_-Br], (**d**) PA-*g*-[(PMMA_164_-H)-*mixed brushes*-PDMAEMA_63_-Br].

The effectiveness of the fiber modification was also confirmed by contact angle measurements. The sessile drop was used as the standard arrangement for optical measurement of the contact angle using shape analysis. In the case of the static contact angle, the deposited drop lies on the surface of a fiber. Distilled water was used as the liquid. In the case of unmodified fibers, the water drops immediately soaked into the fabric and the contact angle (CA) values were close to 0° and are not shown. After modification with PMMA and PS polymer brushes, the surface turned hydrophobic and the contact angles dramatically increased to 118° and 115°, respectively. Images of water drops placed on the surface are provided in Figure 2. There was no significant difference in CA values between PA-*g*-PMMA_164_-Br and PA-*g*-PS_66_-Br because of the nature of the grafted polymer. Although the contact angles values were not correlated with the local texture of the fabric, the dramatic differences between unmodified and modified fibers confirm the polymer growth on the fibers’ surfaces [39,40,41].

The PA-*g*-PMMA_164_-Br or PA-*g*-PS_66_-Br modified knitted fabric were boiled in water for 1 h. Fibers were similar to organic or inorganic particles due to the fact of their chemical character and spontaneously acquired a surface electrical charge when brought into contact with a polar medium such as water. Moreover, the glass temperature of polyamides varied from 40 °C to 70 °C; therefore, in parallel, the thermal rearrangement of polymer chains was expected to take place, moving the free amine and acidic groups to the fiber surface which is in contact with water molecules [42]. Such rearrangement allowed saturation of the fibers’ surfaces with amine free chain end groups to be used for further ATRP initiator immobilization. Also, since both PMMA and PS are hydrophobic, they contracted in water, limiting the contact of the surface of the fibers with this solvent, leading to the better exposure of PA chains ends on the surface to the solvent environment. Prior to the second initiator immobilization, chains of PMMA and PS already tethered to the fibers surface were deactivated by Bu_3_SnH or TEMPO radicals in order to prevent the polymer growth by ATRP from already existing chains [43,44]. The yielded PA-*g*-PMMA_164_-H and PA-*g*-PS_66_-TEMPO fabrics were analyzed by EA and confirmed no bromine atoms were present within samples. Therefore, such hybrids were modified once again with ATRP-initiating groups, BIBB, resulting in PA-*g*-[(PMMA_164_-H), Br] and PA-*g*-[(PS_66_-TEMPO), Br], respectively. Since the bromine content on the modified surface determined by elemental analysis was again very low, reaching 0.28% wt and 0.2% wt, the molar ratios among all reagents used for the SI-ATRP were calculated according to the amount of free initiator only. In parallel the SEM-EDS analysis of fibers surface before and after modification with 2-bromo-2-methylpropionyl bromide, confirmed qualitatively that bromide occurred on the surface of nylon fiber after modification (Figures S6–S9).

In contrast to the hydrophobic polymer of PMMA and PS, the hydrophilic polymer poly((2-dimethylamino)ethyl methacrylate) (PDMAEMA) was grafted from the surface in the second step of ATRP polymerization. With the MW-dependent lower critical solution temperature (LCST) in the range of 40–46 °C, PDMAEMA offers a wide range of applications and thermal responsiveness [45]. The DMAEMA was polymerized from the surface and in solution using the following molar ratio of reagents: DMAEMA/EBIB/CuBr/CuBr_2_/dNbpy = 300/1/0.5/0.5/2. The polymerizations proceeded with good control, and the GPC results of PDMAEMA synthesized in solution are shown in Table 1 and Figure S5. The mass of modified textiles increased to 2.5 ± 0.2 mg and 3.1 ± 0.2 mg, and corresponded to 0.75% wt and 0.85% wt of mass change for PA-*g*-[(PMMA_164_-H)-*mixed brushes*-PDMAEMA_63_-Br] and PA-*g*-[(PS_66_-TEMPO)-*mixed brushes*-PDMAEMA_119_-Br], respectively.

**Scheme 1:**
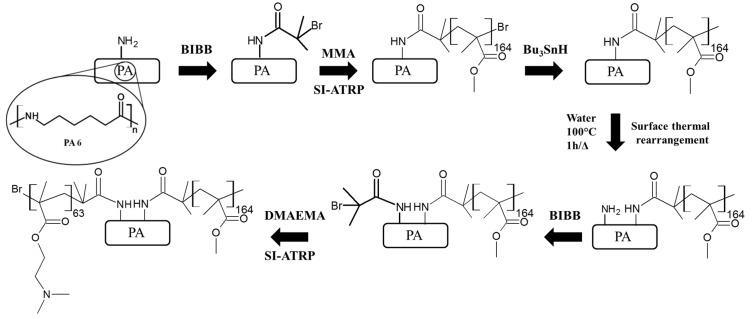
The synthetic pathway for the surface modification of polyamide (PA) fibers by poly(methyl methacrylate) (PMMA) and poly((2-dimethylamino)ethyl methacrylate) (PDMAEMA) homopolymers; BIBB - 2-bromo-2-methylpropionyl bromide; SI-ATRP - surface-initiated atom transfer radical polymerization.

The effective grafting of PDMAEMA on PA fibers containing already tethered chains of PMMA and PS was verified by the FTIR spectroscopy. Figure 1a, b presents the spectra of PA fibers with grafted PMMA or PS and PDMAEMA, respectively. The presence of the peak at 2750 cm^−1^ is assigned to the presence of -N(CH_3_)_2_ group from PDMAEMA [36]. Contact angle values changed from 118° to 105° and 115° to 104°, respectively. The decreased contact angle values indicated that the hydrophobicity of the surface can be tuned by the second step polymerization with hydrophilic PDMAEMA chains. Scheme 1 illustrates the synthetic pathways of PA surface modification with mixed polymer brushes. 

**Figure 3:**
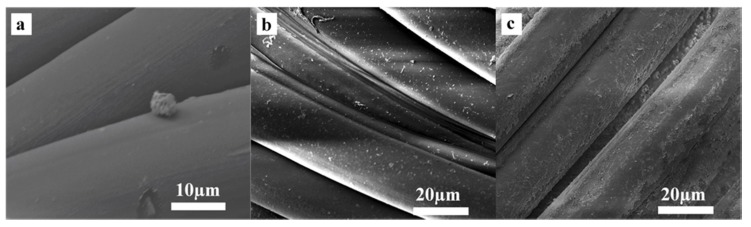
SEM images of PA fibers (**a**), modified by PA-*g*-PS_66_-Br (**b**), and PA-*g*-[(PS_66_-TEMPO)-*mixed brushes*-PDMAEMA_119_-Br] (**c**).

The SEM images of the fibers’ surface before and after the tethering of PS and PDMAEMA are shown in Figure 3. Pure PA fibers presented a smooth surface (Figure 3a). The roughness of the fiber surface increased after modification; either granules or glutinous substances were observed on the surface (Figure 3b,c). The SEM images confirmed the changes of surface morphology after polymer brushes were grafted. However, it was impossible to determine the exact thickness of the polymer film through this analysis. 

**Figure 4:**
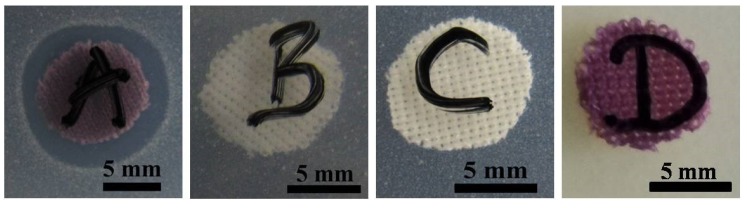
Images of bacteriostatic properties of the PA surface (**a**) modified with tethered gold nanoparticles Au (NPs) @PA-*g*-[(PMMA_164_-H)-*mixed brushes*-PDMAEMA_63_-Br], (**b**) unmodified PA, (**c**) modified with tethered PA-*g*-[(PMMA_164_-H)-*mixed brushes*-PDMAEMA_63_-Br], and (**d**) PA with soaked Au NPs.

The surface tethered polymer chains offer the possibility of further modifications using polymer functional groups of the modified fibers. Herein, the PDMAEMA chains were used as loading sites for gold nanoparticles (Au NPs). Gold NPs were immobilized within the grafted polymer layer through electrostatic interaction between the amine groups and the loaded gold precursor, AuCl_4_^−^, followed by the in situ reduction to yield Au NPs@PA-*g*-[(PMMA_164_-H)-*mixed brushes*-PDMAEMA_63_-Br]. Consequently, the yielded fibers turned purplish in color which is consistent with the typical Au NPs plasmonic color, demonstrating the successful *in situ* synthesis of Au NPs (Figure 4). Since the Au NPs could be used as bioactive materials, the antibacterial activity of Au NPs@PA-*g*-[(PMMA_164_-H)-*mixed brushes*-PDMAEMA_63_-Br] was analyzed by estimation the resistance of growth for *E. coli* reflected by the formation of inhibition halo around the sample [46]. In comparison to unmodified PA, PA-*g*-[(PMMA_164_-H)-*mixed brushes*-PDMAEMA_63_-Br], and a blank sample of PA loaded with Au NP (Figure 4b–d), which showed no antibacterial activity, the growth of *E. coli* in the case of Au NPs@PA-*g*-[(PMMA_164_-H)-*mixed brushes*-PDMAEMA_63_-Br] was significantly inhibited in the zone with a diameter of 2 mm (Figure 4a). Therefore, it can be concluded the modified fibers can be easily designed with antibacterial activity. Such an approach may pave the way for future application in medicine. 

## 4. Conclusions

In summary, it was possible to subsequently graft two different types of polymer brushes from the surfaces of polyamide fibres using atom transfer radical polymerization due to the utilization of the PA chains’ end groups for the attachment ATRP initiators. The amount of the PA chains’ end groups available on the modified surface was tuned by thermal surface rearrangement. This was a totally new approach in the synthesis of tethered mixed polymer brushes. An analysis of the bactericidal properties demonstrated that the surface properties could be easily designed, simply by choosing the functionality of tethered polymers. Thanks to the simplicity of the presented approach, it is expected that this strategy will be further developed to control the density and character of the grown polymer chains. Therefore, the surface properties may be precisely tuned, leading to new applications.

## Figures and Tables

**Table 1:** Characteristics of free polymers PMMA, PS, and PDMAEMA.

Entry	Sample	*M*_theor_^a^	*M_n_* ^b^	*M_w_*/*M_n_*
1	PA-*g*-PMMA_164_-Br	16,400	17,400	1.11
2	PA-*g*-[(PMMA_164_-H)-*mixed brushes*-PDMAEMA_63_-Br]	9400	11,600	1.13
3	PA-*g*-PS_66_-Br	7000	7500	1.18
4	PA-*g*-[(PS_66_-TEMPO)-*mixed brushes*-PDMAEMA_119_-Br]	20,600	17,900	1.19

^a^ [*M*] The theoretical molecular weight (*M*_theor_) was calculated based on ^1^H NMR. ^b^ [*M*] The *M_n_* and *M_w_*/*M_n_* were determined by GPC (PS or PMMA standards).

## Supplementary Materials

The following are available online at https://www.mdpi.com/2079-4991/10/3/536/s1.
