# Supplementary material for: Renewable Fabric Surface-Initiated ATRP Polymerizations: Towards Mixed Polymer Brushes
Source: Nanomaterials (Basel). 2020 Mar 17;10(3):536. doi: 10.3390/nano10030536 (PMC7153387; doi:10.3390/nano10030536)

Supporting Information for

Renewable Fabric Surface Initiated ATRP Polymerizations: towards Mixed Polymer Brushes

Wojciech Raj[1], Alessandro Russo[2], Yaoming Zhang[1,3], Julien Chapelat[2] and Joanna Pietrasik[1*]

[1] Lodz University of Technology, Institute of Polymer and Dye Technology, Stefanowskiego 12/16, 90-924 Lodz, Poland
[2] Cemex Research Group AG, Römerstrasse 13, 2555 Brüggbei Biel, Switzerland
[3] Key Laboratory of Solid Lubrication, Lanzhou Institute of Chemical Physics, Chinese Academy of Sciences, Lanzhou 730000, China
* Correspondence: joanna.pietrasik@p.lodz.pl

EXPERIMENTAL SECTION

Modification of PA knitted fabric; PA-*g*-[(PS_{66}-TEMPO)-*mixed brushes*-$PDMAEMA_{119}$-Br]

PA modification with ATRP initiator; PA-Br

PA knitted fabric (5.0 g), *N*-methyl-2-pyrrolidone (50.0 mL) and triethylamine (4.3 mL, 30.99 mmol), were added to a round bottom flask and then it was immersed in an ice bath. After the flask cooled, 2-bromo-2-methylpropionyl bromide BIBB (4.6 mL, 37.2 mmol) was added drop-wise over ~ 30 min. The reaction mixture was stirred for 24 h at room temperature. The modified fibers were removed from reaction mixture and washed several times with acetone to obtain the PA fabric with tethered ATRP initiators, PA-Br.

Grafting of polystyrene from PA surface via SI-ATRP; PA-*g*-PS_{66}-Br

A Schlenk flask was charged with modified knitted fabric (PA-Br) (423.5 mg), copper(I) bromide (29.2 mg, 0.204 mmol), copper(II) bromide (19.5 mg, 0.087 mmol) and 4,4′-dinonyl-2,2′-dipyridyl (237.8 mg, 0.582 mmol). Next, the flask was degassed by purging with argon for 45 min at room temperature. Styrene (10.0 mL, 87.3 mmol), anisole (2.5 mL) and ethyl 2-bromo-2-methylpropionate EBIB (42.7 μL, 0.291 mmol) were degassed separately and added via a syringe to the reaction flask. The flask was placed in an oil bath heated to 80°C for 48 h. The polymerization was stopped by opening the flask and exposing the reaction mixture to air. The obtained knitted fabric was separated from the reaction mixture, washed by extraction with acetone and used for further analysis. The free polystyrene was dissolved in tetrahydrofuran THF, purified by passing through a chromatography column filled with neutral alumina, precipitated and dried in the vacuum oven.

Deactivation of PA-*g*-PS_{66}-Br chain-end with 2,2,6,6-tetramethyl-1-piperidinyloxy TEMPO; PA-*g*-PS_{66}-TEMPO

Polystyrene modified knitted fabric PA-*g*-PS_{66}-Br (380.2 mg), CuBr (28.4 mg, 0.198 mmol), 2,2,6,6-tetramethyl-1-piperidinyloxy TEMPO (61.8 mg, 0.396 mmol) and 4,4′-dinonyl-2,2′-dipyridyl (80.9 mg, 0.198 mmol) were put into a Schlenk flask. Next, the flask was degassed by purging argon for 45 min at room temperature and then degassed acetone (20.0 mL) was added via a syringe. The reaction to replace the–Br end group by TEMPO was started after putting the flask into an oil bath heated to 70° C, and was complete after 12 h reaction. The resulting knitted fabric PA-*g*-PS_{66}-TEMPO was washed a few times with acetone and dried in vacuum oven over night.

Rearrangement of PA-*g*-PS_{66}-TEMPO

Obtained fabric PA-*g*-PS_{66}-TEMPO (350.0 mg) and deionised water (100 mL) were put into a beaker and heated under water boiling conditions. The knitted fabric was removed from the water after 1 h and dried in a vacuum oven for 24 h.

Modification PA-*g*-PS_{66}-TEMPO with an ATRP initiator; PA-*g*-[(PS_{66}-TEMPO), Br]

Knitted fabric PA-*g*-(PS_{66}-TEMPO) (350 mg), *N*-methyl-2-pyrrolidone (30.0 mL) and triethylamine (0.34 mL, 2.45 mmol) were added to a flask immersed in an ice bath. Once the contents of the flask were cooled down, 2-bromo-2-methylpropionyl bromide (0.36 mL, 2.95 mmol) was added drop-wise over ~ 30 min. The reaction mixture was kept at room temperature for 12 h. The modified fibers were removed from the reaction mixture and washed several times with acetone and dried in vacuum oven overnight.

Synthesis of mixed-polymer brushes; PA-*g*-[(PS_{66}-TEMPO)-*mixed brushes*-$PDMAEMA_{119}$-Br]

A Schlenk flask was charged with modified PA-*g*-[(PS_{66}-TEMPO), Br] knitted fabric (364.3 mg), copper(I) bromide (14.2 mg, 0.099 mmol), copper(II) bromide (22.1 mg, 0.099 mmol) and 4,4′-dinonyl-2,2′-dipyridyl (161.7 mg, 0.396 mmol). Next, the flask was degassed by purging with argon for 45 min at room temperature. 2-(Dimethylamino)ethyl methacrylate DMAEMA (10.0 mL, 59.35 mmol), acetone (5.0 mL) and ethyl 2-bromo-2-methylpropionate EBIB (29.0 μL, 0.198 mmol) were degassed separately and added via a syringe to the reaction flask. The flask was placed in an oil bath heated to 60° C for 8 h. The obtained knitted fabric was separated from the reaction mixture, washed by extraction with acetone and used for further analysis. Free PDMAEMA was dissolved in THF, purified by passing through a chromatography column filled with neutral alumina, precipitated and dried in the vacuum oven.

Figure S1. ^{1}H NMR spectra collected in $CDCl_3$ (250 MHz) during polymerization of methyl methacrylate MMA from PA-Br; PA-*g*-$PMMA_{164}$-Br

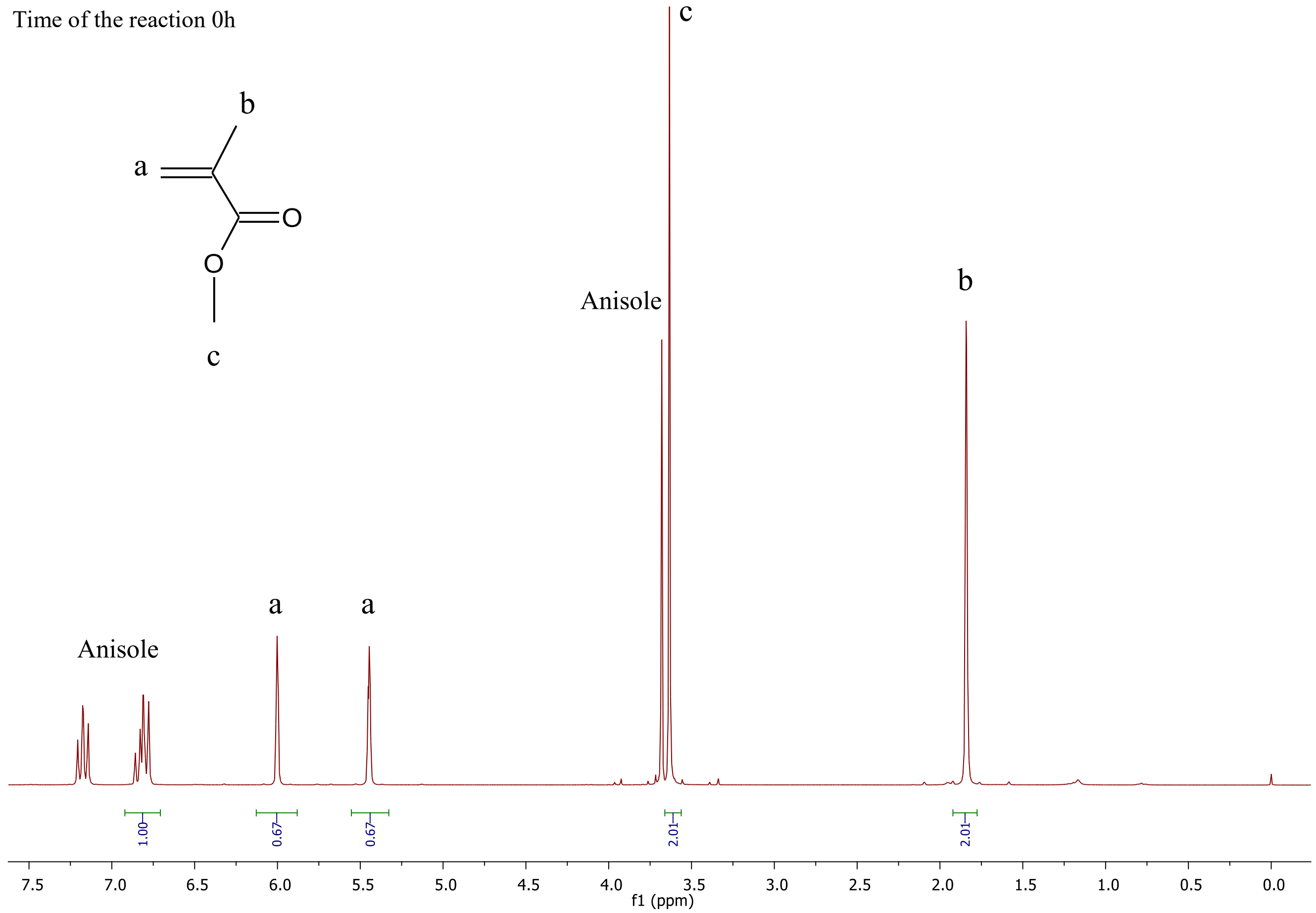

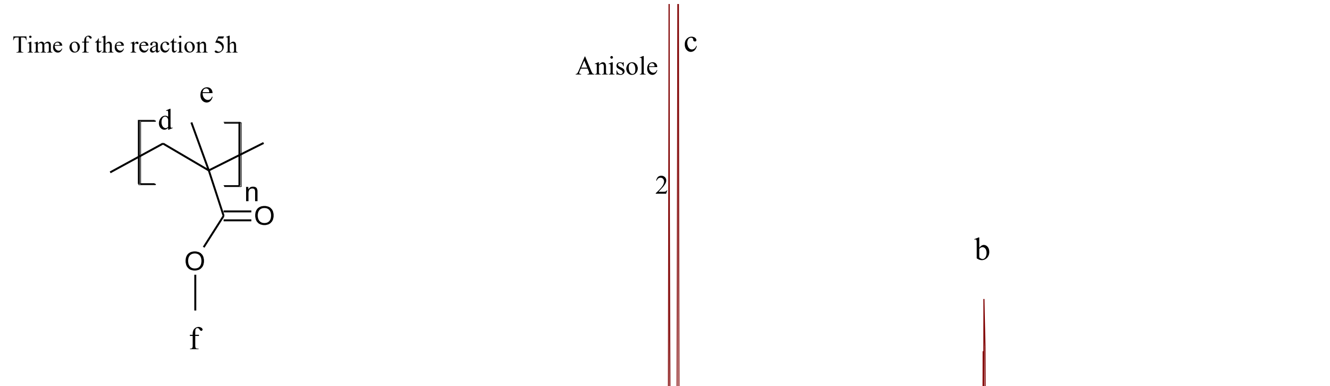

Figure S2. ^{1}H NMR spectra collected in $CDCl_3$ (250 MHz) during polymerization of 2-(dimethylamino)ethyl methacrylate DMAEMA from PA-*g*-[($PMMA_{164}$-H), Br]; PA-*g*-[($PMMA_{164}$-H)-*mixed brushes*-$PDMAEMA_{63}$-Br]

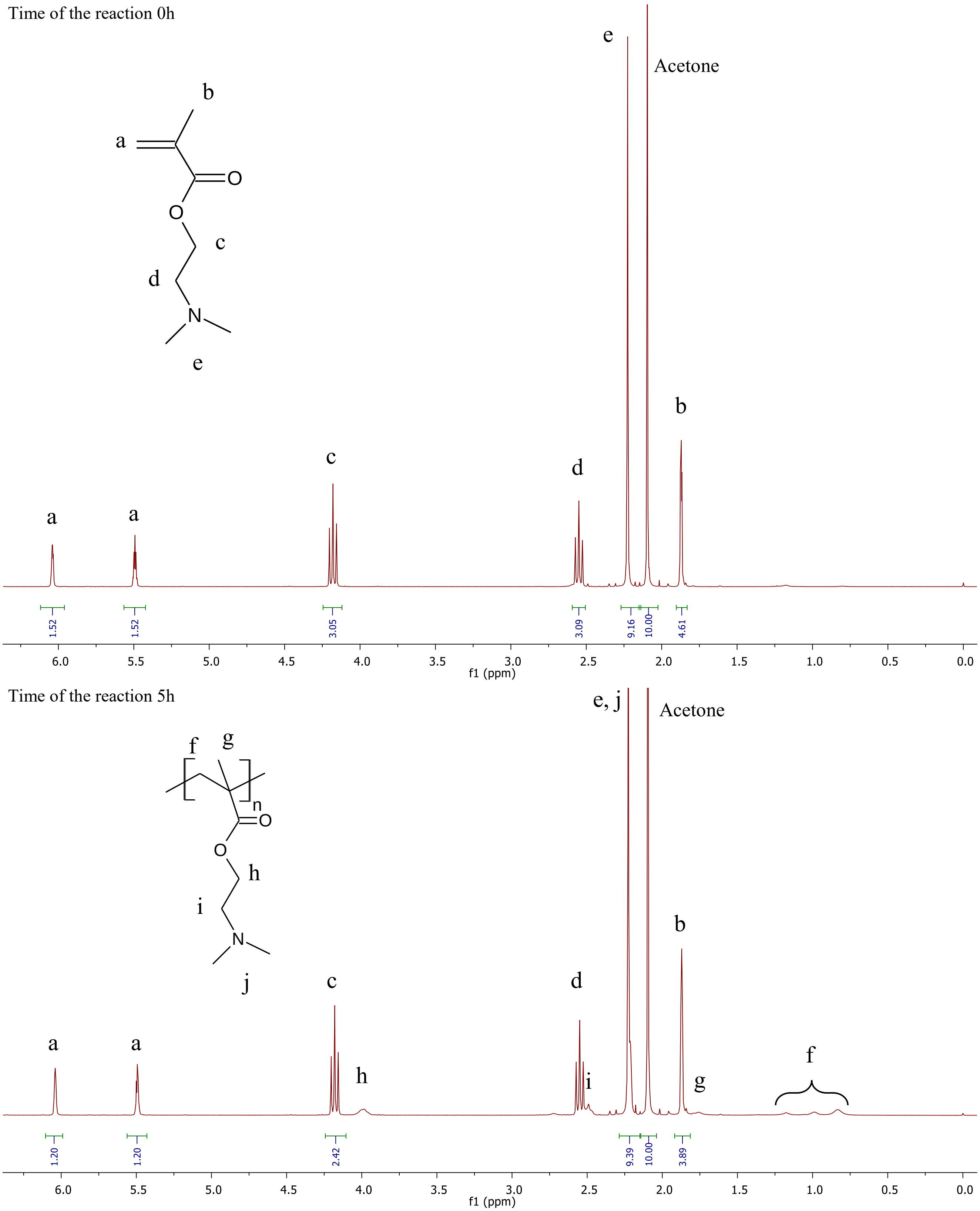

Figure S3. ^{1}H NMR spectra collected in $CDCl_3$ (250 MHz) during polymerization of styrene S from PA-Br; PA-*g*-PS_{66}-Br

Time of the reaction 0h

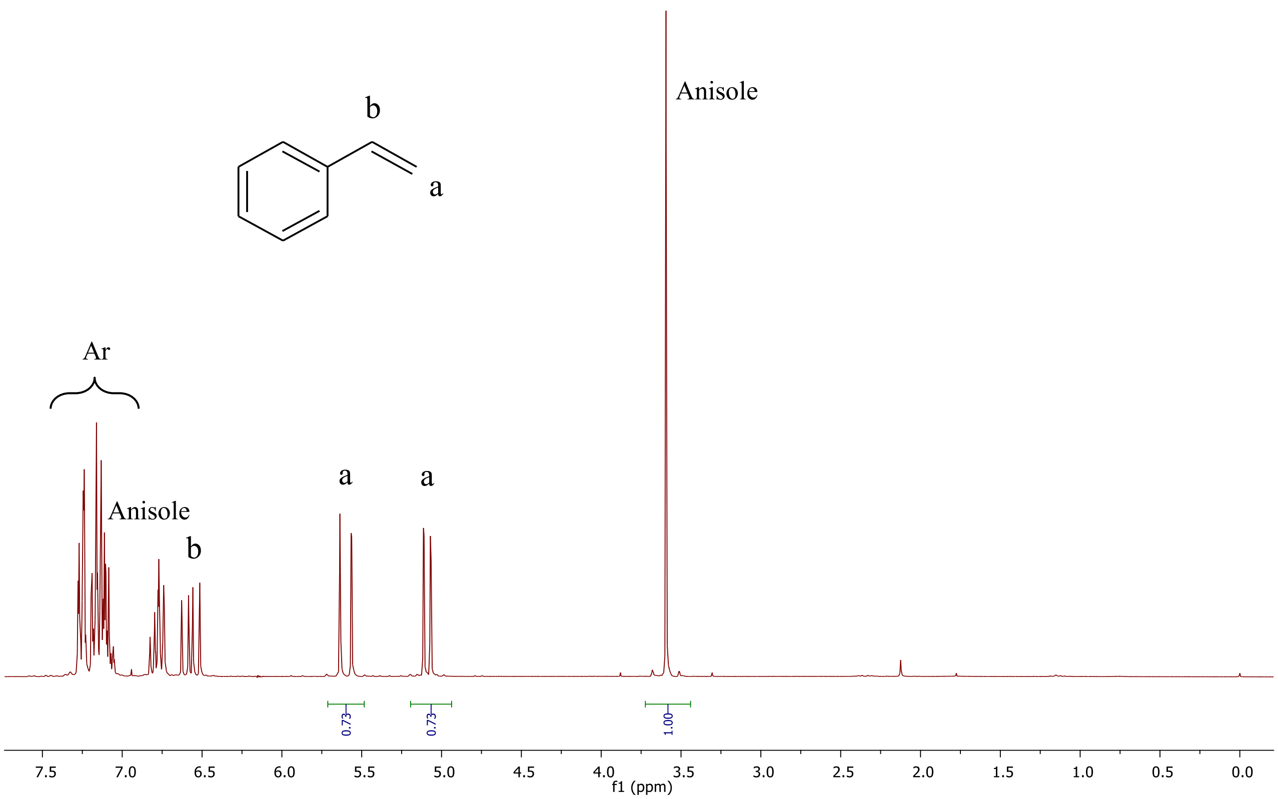

Time of the reaction

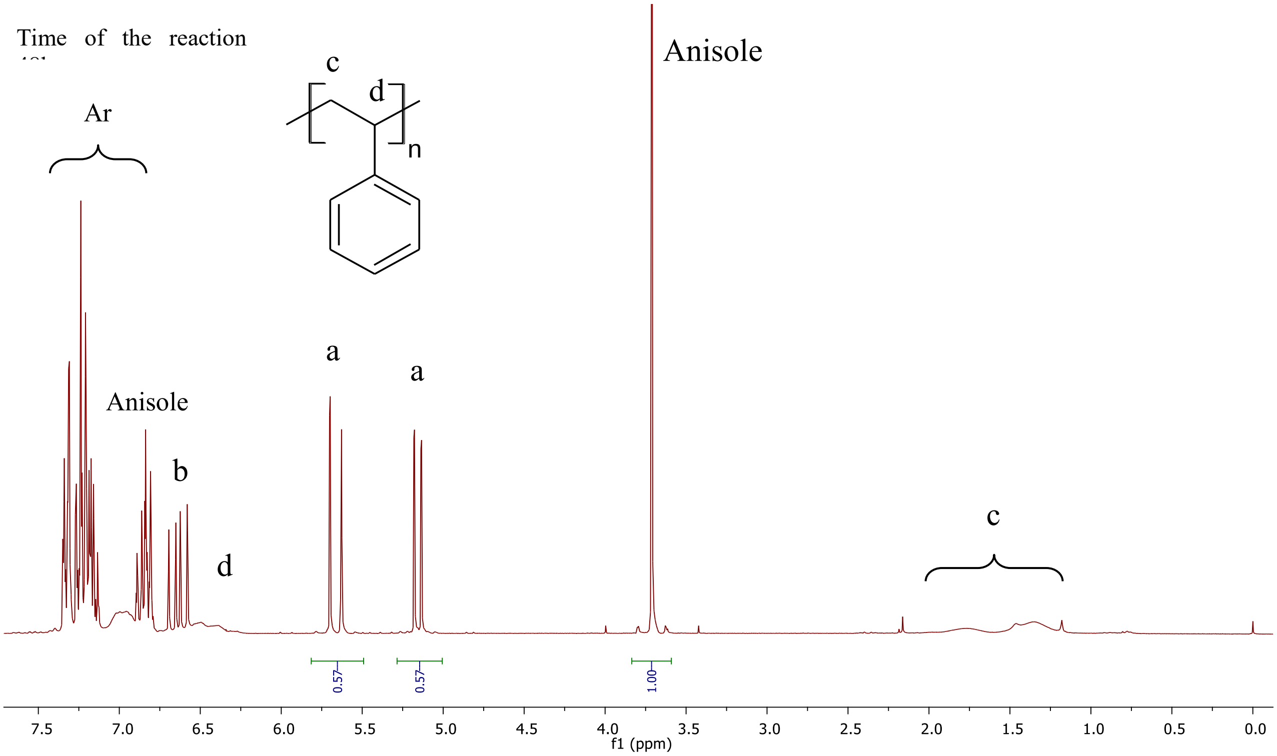

Figure S4. ^{1}H NMR spectra collected in $CDCl_3$ (250 MHz) during polymerization of 2-(dimethylamino)ethyl methacrylate DMAEMA from PA-*g*-PS_{66}-TEMPO; PA-*g*-[(PS_{66}-TEMPO)-*mixed brushes*-$PDMAEMA_{119}$-Br]

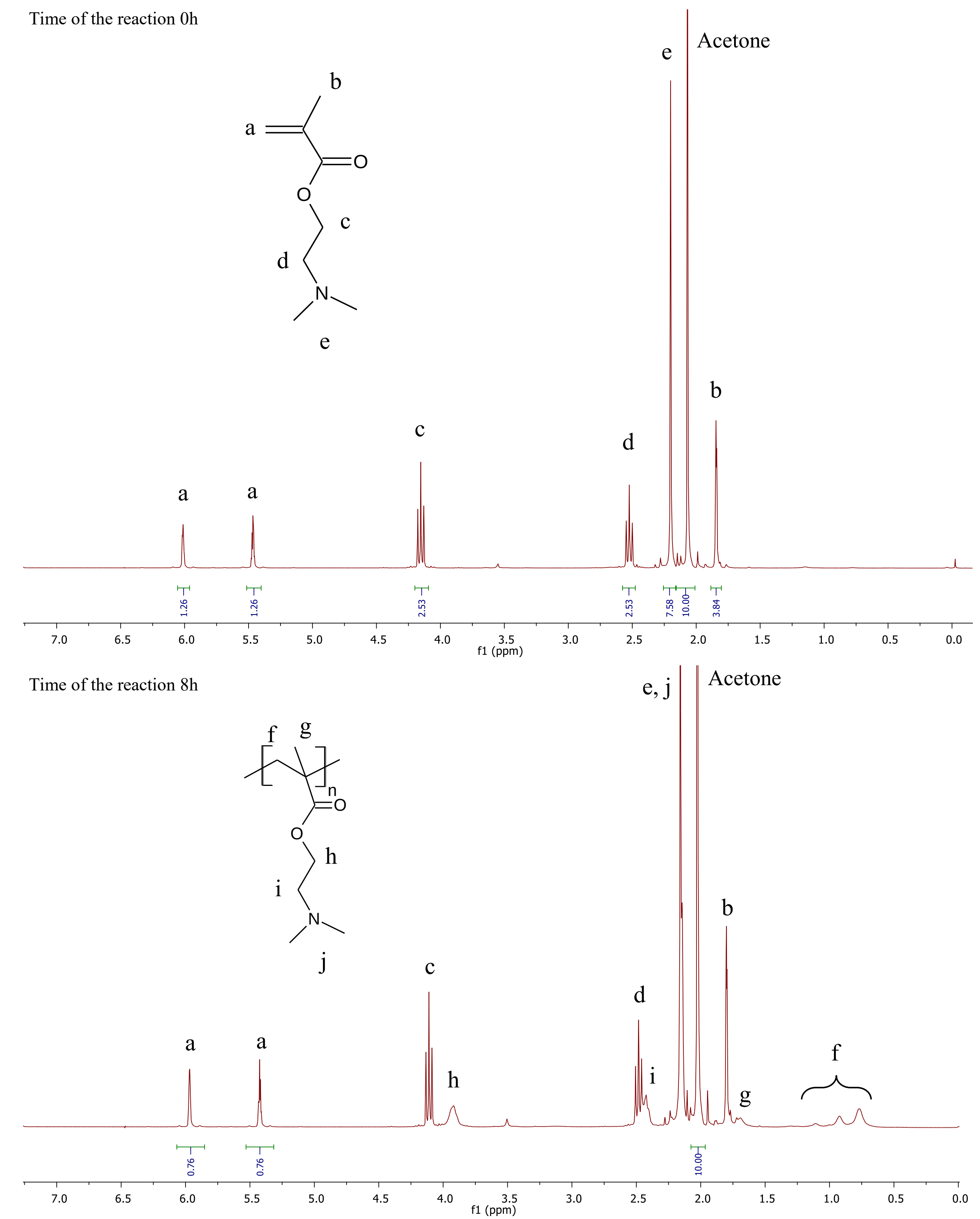

Figure S5. Gel permeation chromatography GPC traces of free polymers PMMA, PS and PDMAEMA synthesized in solution.

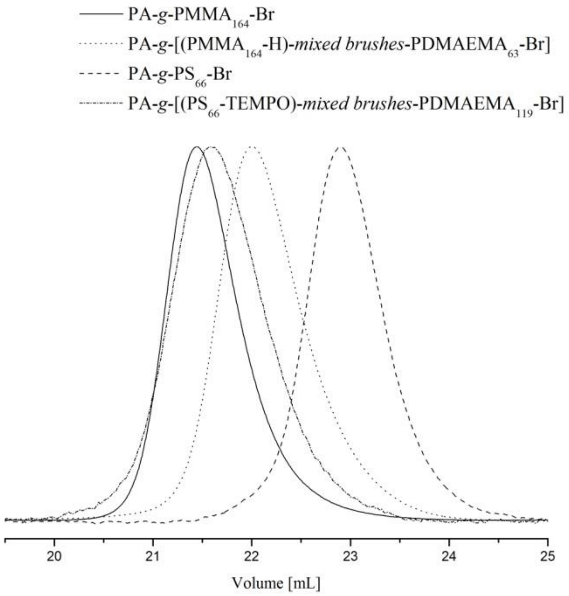

Figure S6. Scanning electron microscopy – energy-dispersive spectroscopy SEM – EDS spectrum of pure polyamide fabric; PA

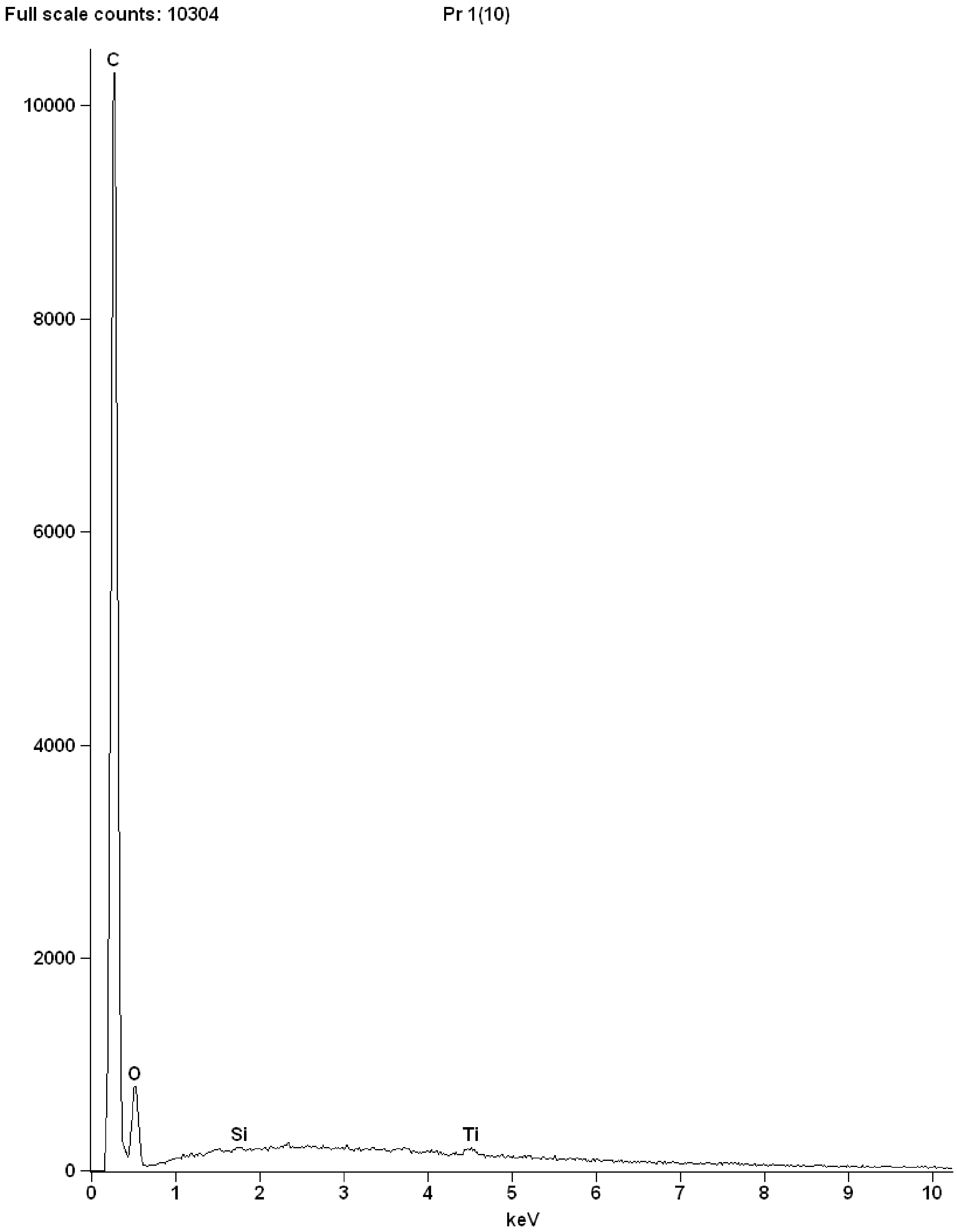
Full scale counts: 10304
Pr 1(10)
C
O
Si
Ti
10000
8000
6000
4000
2000
0
0
1
2
3
4
5
6
7
8
9
10
keV

Figure S7. SEM – EDS spectrum of polyamide fabric modified with 2-bromo-2-methylpropionyl bromide; PA-Br

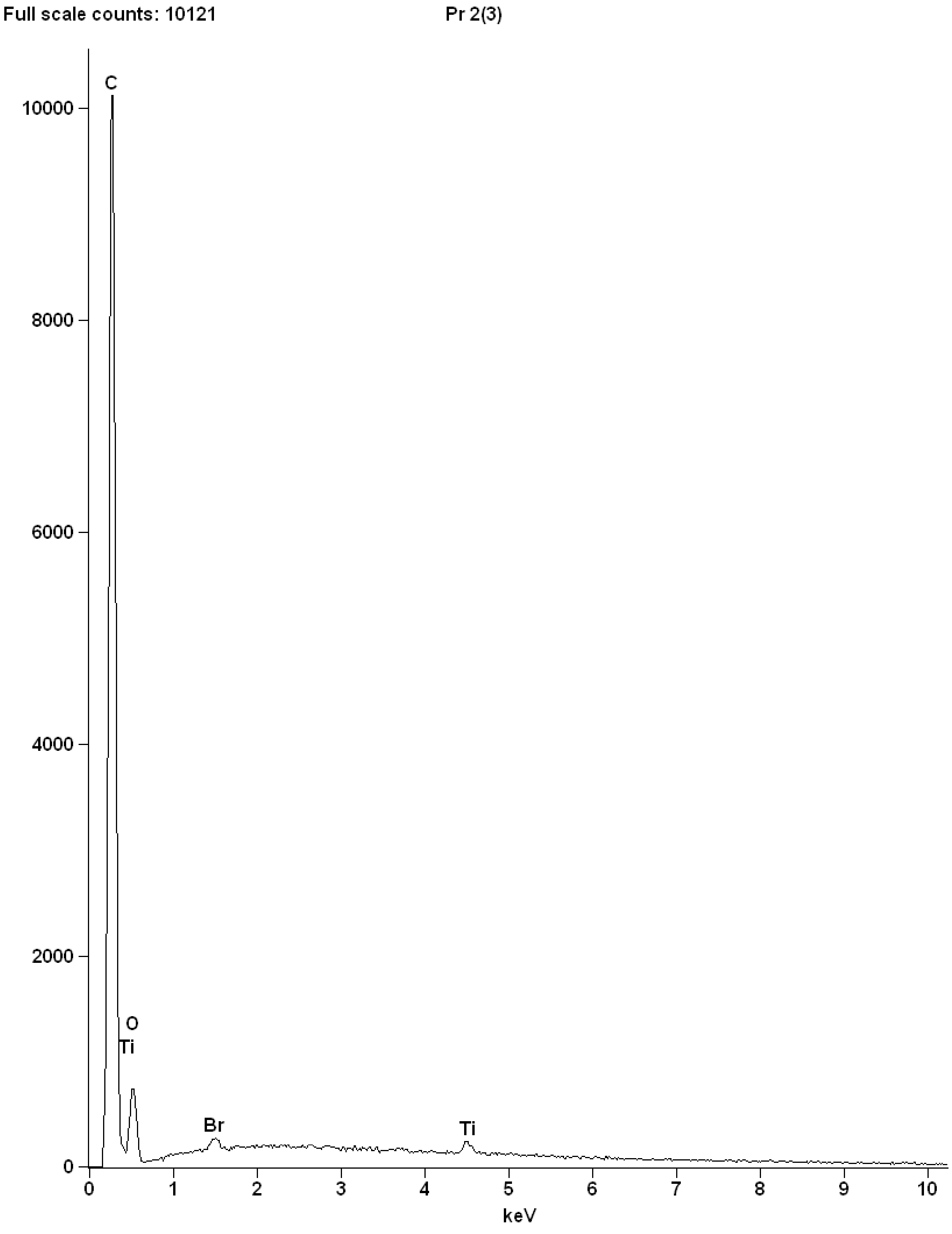

Figure S8. SEM – EDS spectrum of modified polyamide fabric PA-*g*-$PMMA_{164}$-Br after chains ends deactivation by tributyltin hydride Bu_3SnH; PA-*g*-$PMMA_{164}$-H

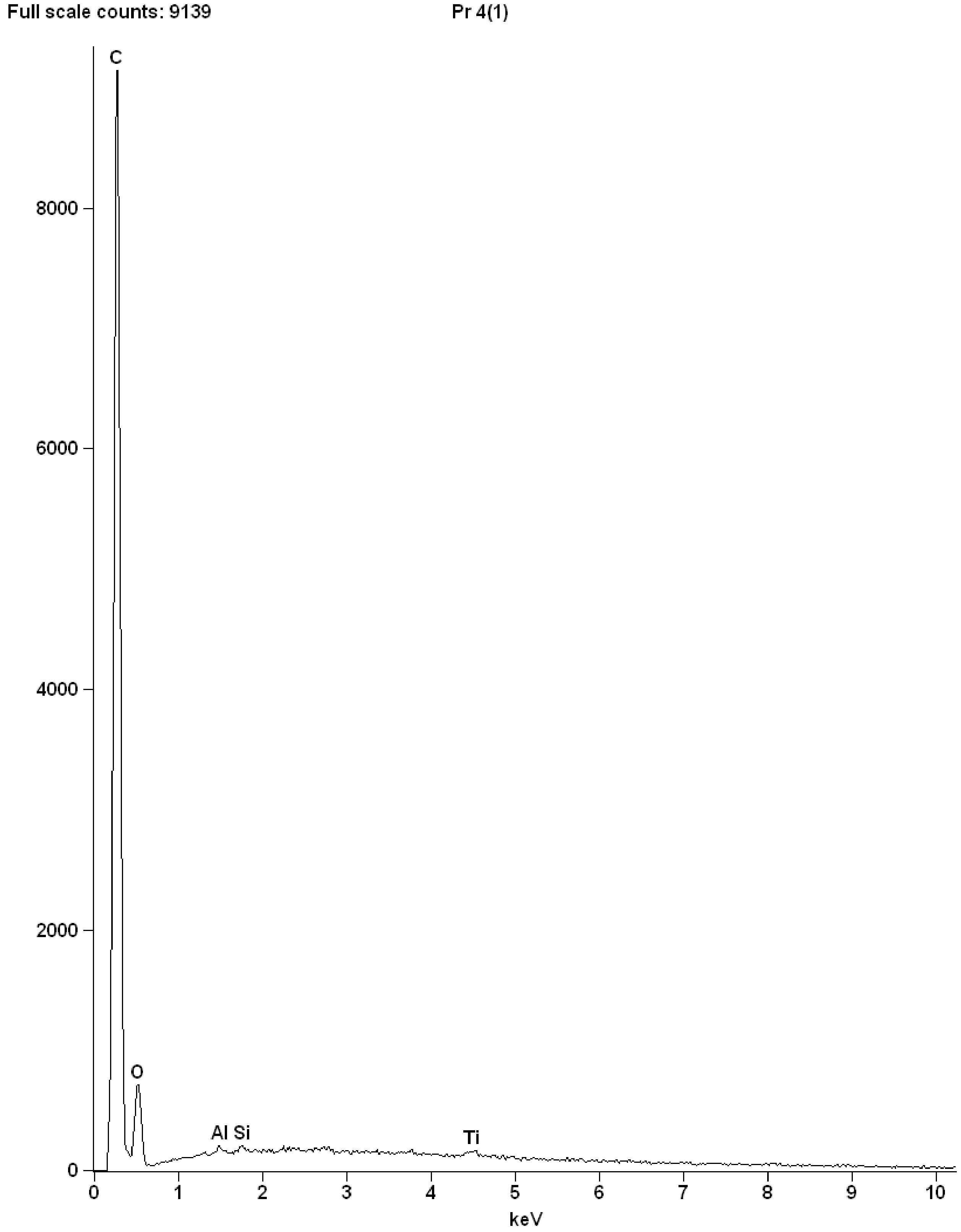

Figure S9. SEM – EDS spectrum of modified polyamide fabric after second modification with 2-bromo-2-methylpropionyl bromide; PA-*g*-[(PMMA$_{164}$-H), Br]

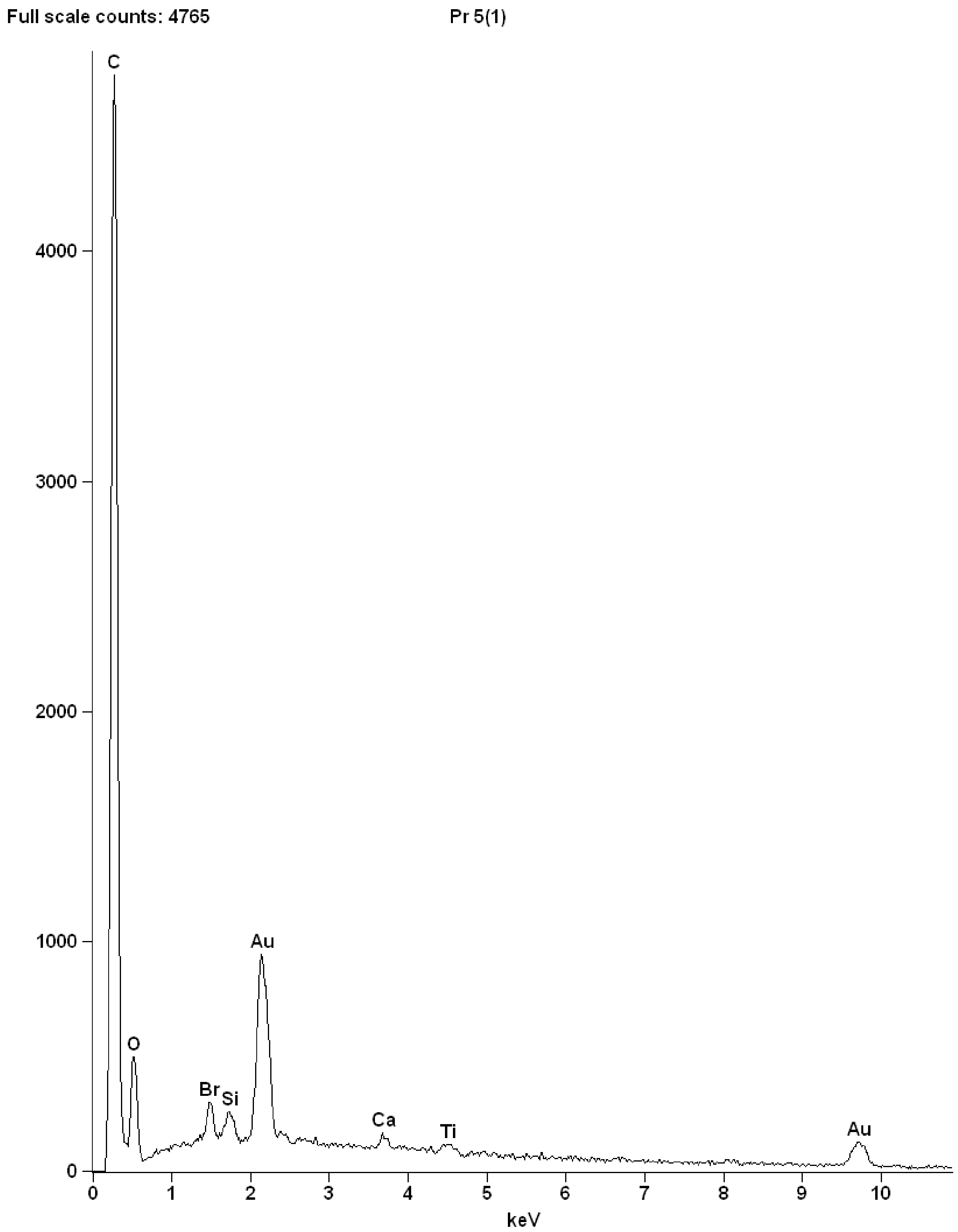

Supplement: Supplementary file 1 [file nanomaterials-10-00536-s001.pdf]
